# Machine learning prediction of acute myocardial infarction incidence using regional meteorological data

**DOI:** 10.3389/fcvm.2026.1678649

**Published:** 2026-03-13

**Authors:** Chao Li, Lin Mu, Hongyan Zhu, Guangyao Zhai

**Affiliations:** 1 Department of Cardiology, Beijing Luhe Hospital, Capital Medical University, Beijing, China; 2 Endocrine and Rheumatology Centre, Beijing Luhe Hospital, Capital Medical University, Beijing, China

**Keywords:** acute myocardial infarction, diurnal temperature range, machine learning, meteorological prediction, resource allocation

## Abstract

**Background:**

Acute myocardial infarction (AMI) is significantly influenced by meteorological conditions; however, leveraging meteorological data to predict AMI incidence remains challenging. This study aimed to analyze weather-AMI associations and construct a predictive model using machine learning.

**Methods:**

We conducted a retrospective analysis of AMI patients from a regional chest pain center, coupled with local daily weather data spanning 10 years. The relationship between weather variables and daily AMI case counts was analyzed. A Random Forest model was employed to capture potential non-linear relationships. Model performance was validated using real-world data.

**Results:**

Over 4,197 days (January 2013–June 2024), 11,527 AMI patients were included. Days with higher AMI incidence were characterized by lower temperatures, greater daily temperature differences (*Δ*T), and reduced air speed, while exhibiting lower humidity and precipitation compared to days with fewer cases. After multivariable adjustment, daily *Δ*T and air speed showed statistically significant associations with increased AMI incidence (*p* < 0.05), whereas other weather elements did not. In the predictive model, daily *Δ*T emerged as the most important factor. Validation demonstrated moderate discriminative ability, with an AUC-ROC of 0.67 (95% CI: 0.60–0.74). Sensitivity analysis using the 95th percentile as a threshold further confirmed the model's effectiveness.

**Conclusions:**

This study identifies daily temperature variation (*Δ*T) and air speed as significant meteorological predictors of AMI incidence. The Random Forest model effectively captured non-linear weather-AMI relationships, supports integrating weather data—particularly temperature variability—into AMI risk stratification systems. Future research should enhance predictive power by incorporating clinical and demographic variables alongside environmental factors.

## Background

Acute myocardial infarction (AMI) represents a leading global cause of mortality, with reported incidence rates ranging from 40 to 300 per 100,000 population across studies (1–5). Early reperfusion therapy, the preferred intervention for AMI patients (6), significantly improves clinical outcomes (7). Timely administration of this treatment necessitates adequate allocation of medical resources, as insufficient capacity may compromise optimal therapeutic timing.

Environmental factors substantially influence AMI occurrence, particularly temperature variability and air pollution. Short-term exposure to severe air pollution elevates AMI risk (8–10), while both absolute temperatures and temperature fluctuations correlate with infarction events (11–13). Notably, extreme temperatures may increase hospitalization rates for AMI, though regional and ethnic differences in weather sensitivity exist (14, 15). While prior research has predominantly examined weather-AMI associations, few studies have explored using meteorological parameters to optimize AMI treatment resource allocation.

Machine learning (ML) algorithms offer advantages in handling complex datasets and uncovering non-linear patterns, frequently outperforming traditional predictive models (16, 17). These techniques show growing utility in medical applications, demonstrating efficacy in imaging interpretation, disease risk prediction, and clinical decision support (18–20). Notably, in environmental health, ML models like Long Short-Term Memory networks have been successfully applied to predict infectious disease outbreaks, such as dengue fever, by leveraging meteorological variables (21). Furthermore, ML models (e.g., LightGBM) have been employed to quantify the mortality risk of cardiovascular diseases like myocardial infarction attributable to temperature extremes, and to project the increased risk under future climate scenarios using transfer learning (22).

This study aims to: (1) analyze associations between meteorological parameters (temperature, wind speed, humidity, etc.) recorded in Beijing's Tongzhou District and daily AMI patient volume at Luhe Hospital; (2) develop an ML-based prediction model; and (3) evaluate the model's predictive performance using real-world data.

## Methods

### Study design and data access

To analyze the impact of meteorological factors on acute myocardial infarction (AMI) incidence, we obtained daily weather records (January 2013–June 2024) from the Tongzhou District Meteorological Observatory (Station ID: 54431) east of Beijing and corresponding daily AMI admission records from Beijing Luhe Hospital. Tongzhou District, with a stable resident population of approximately 1.5 million (average annual growth rate <5%), designates Beijing Luhe Hospital as its primary chest pain center. This facility receives most emergency intervention-eligible AMI patients in the region.

Weather data—including daily mean/maximum/minimum temperatures, humidity, precipitation, and wind speed—were sourced from China's National Meteorological Science Data Center (https://data.cma.cn). Daily meteorological records, including mean, maximum, and minimum temperature, relative humidity, precipitation, and wind speed, were obtained for the study period. To quantify relevant weather variations, the following indicators were calculated:

Diurnal temperature range (°C): defined as the difference between the daily maximum and minimum temperature.
Expected temperature difference (°C): defined as the difference between the next day's mean temperature and the current day's mean temperature.
Expected humidity difference (%): calculated as the next day's mean relative humidity minus the current day's mean relative humidity.
Expected precipitation difference (mm): calculated as the next day's total precipitation minus the current day's total precipitation.
Expected wind speed difference (m/s): calculated as the next day's mean wind speed minus the current day's mean wind speed.

These “expected difference” indicators reflect the day-to-day change in each meteorological variable and were included to examine whether short-term weather shifts were associated with AMI admissions.

Daily AMI patient volumes were extracted from the hospital's Health Information System (HIS) using ICD diagnostic codes.

## Statistical analysis and predictive modeling

Based on the distribution of daily case counts, we defined two distinct prediction targets: 1) Daily AMI Case Count: a continuous variable representing the exact number of admissions each day; 2) High-Incidence Day: a binary variable where days with case counts at or above the 90th percentile (≥5 cases) were classified as “High-Incidence,” and all others as “Normal-Incidence.”

Meteorological factors were compared between High- and Normal-Incidence days using Student's t-test or Pearson's *χ*² test, as appropriate. The relationship between daily AMI number (continuous) and meteorological factors was assessed using multiple linear regression.

To build a predictive model for High-Incidence Days, we adapted Lasso Regression, Random Forest, and Support Vector Regression (SVR) for binary classification. Using the caret package in R, the dataset was randomly partitioned into a training set (75%) for model development and hyperparameter tuning, and a held-out testing set (25%) for final performance evaluation. The model output was the predicted probability of a day being High-Incidence. Model performance was evaluated on the independent test set using the Area Under the Receiver Operating Characteristic Curve (AUC-ROC). Sensitivity analysis was conducted by redefining the classification threshold at the 95th percentile.

## Results

**Figure 1:**
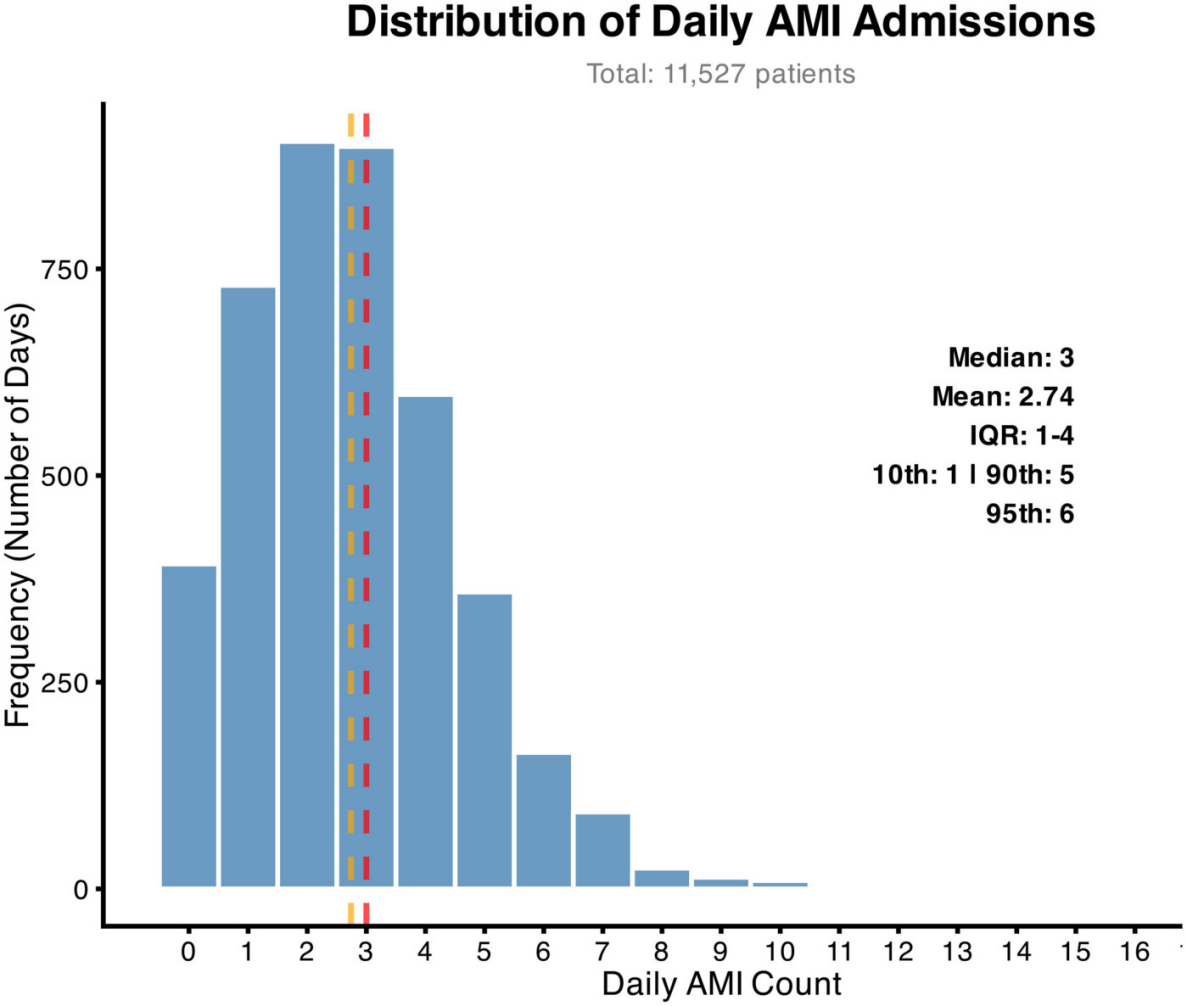
Distribution of the number of patients with myocardial infarction.

From January 2013 to June 2024 (4,197 days), 11,527 AMI patients (including ST-elevated and non-ST-elevated myocardial infarction) were enrolled. As shown in Figure 1, the median daily AMI admissions was 3 (mean: 2.74). The interquartile range spanned from 1 (25th percentile) to 4 (75th percentile), with the 10th and 90th percentiles at 1 and 5, respectively. The 95th percentile was 6. These findings indicate that daily admissions ≤3 cases may indicate adequate resource capacity at this medical center, whereas daily volumes exceeding 5 cases necessitate proactive resource reallocation to ensure timely intervention.

As detailed in Table 1, meteorological records spanning 4,129 days revealed a median daily temperature of 14.2 °C (IQR: 2.5–23.2 °C). The median maximum temperature was 20.6°C (IQR: 8.7–29.1°C), while the median minimum temperature reached 8.3°C (IQR: −2.1–18.2°C). The region exhibited substantial diurnal temperature variability, evidenced by a median daily temperature range of 10.7°C (IQR: 7.9–13.9°C). Precipitation patterns indicated arid conditions, with rainfall occurring infrequently throughout the observation period.

**Table 1:** Baseline characteristics.

Variables	All days *N* = 4,197
Mean temperature (°C)	14.2 (2.5,23.2)
High temperature (°C)	20.6 (8.7, 29.1)
Low temperature (°C)	8.3 (−2.1,18.2)
Daily temperature difference (°C)	10.7 (7.9,13.9)
Relative humidity (%)	58 (41,75)
Precipitation (mm)	0 (0,0)
Air speed (m/s)	2.0 (1.5,2.7)
Temperature Difference (°C)	0.2 (−1.2,1.5)
Expected temperature difference (°C)	−0.2 (−1.5,1.2)
Humidity difference (%)	1.0 (−8.0,10.0)
Expected humidity difference (%)	−1.0 (−10,8.0)
Precipitation difference (mm)	0 (0,0)
Expected precipation difference (mm)	0 (0,0)
Air speed difference (m/s)	0 (−0.6, 0.6)
Expected air speed difference (m/s)	0 (−0.6, 0.6)

As detailed in Table 2, stepwise multiple linear regression revealed a significant positive association between daily temperature difference and daily AMI admissions (*β* = −0.057, *p* < 0.001), and wind speed demonstrated an inverse relationship with AMI incidence (*β* = −0.108, *p* = 0.012).

**Table 2:** Multiple linear regression model of number of AMI patients.

Variables	*P*	*β*	Lower	Higher
Constant	<0.001	2.698	2.469	2.928
Mean temperature	0.091	−0.057	−0.124	0.009
Low temperature	0.263	0.039	−0.029	0.106
Temperature difference	0.004	0.055	0.018	0.093
Wind speed	0.042	−0.055	−0.108	−0.002

As presented in Table 3, stratification by the 90th percentile threshold (≥5 daily AMI cases) classified 669 days as high-incidence periods and 3,530 days as low-incidence periods. High-incidence days exhibited significantly lower ambient temperatures (*p* < 0.01), greater diurnal temperature ranges (*p* < 0.001), and reduced wind speeds (*p* = 0.03) compared to low-incidence days. Conversely, these days demonstrated lower humidity levels (*p* = 0.18) and diminished precipitation (*p* = 0.42), though these differences did not reach statistical significance.

**Table 3:** Comparison of meteorological factors between high incidence group and low incidence group.

Variables	High incidence *N* = 669	Low incidence *N* = 3,530	*p*
Mean temperature	10.7 (0.35, 21.2)	14.5 (2.6,23.3)	<0.001
Maximum temperature	18 (6.95, 27.5)	20.85 (8.8, 29.2)	0.009
Minimum temperature	3.8 (−4.75, 14.8)	8.6 (−1.8, 18.3)	<0.001
Daily temperature difference	12.5 (9.4, 15.3)	10.55 (7.8, 13.8)	<0.001
Relative humidity	54 (39, 71)	58 (42, 75)	0.034
Precipitation	0 (0, 0)	0 (0, 0)	0.998
Air speed	1.9 (1.3, 2.55)	2 (1.5, 2.7)	0.032
Temperature difference	0.3 (−1.1, 1.8)	0.2 (−1.2, 1.4)	0.687
Expected temperature difference	−0.3 (−1.1, 1.2)	−0.15 (−1.5, 1.2)	0.690
Humidity difference	0 (−9, 9)	1 (−8, 10)	0.688
Expected humidity difference	−2 (−10, 7)	−1 (−9, 8)	0.708
Precipation difference	0 (0, 0)	0 (0, 0)	0.881
Expected precipation difference	0 (0, 0)	0 (0, 0)	0.914
Air speed difference	0 (−0.6, 0.6)	0 (−0.6, 0.6)	0.692
Expected air speed difference	0 (−0.6, 0.6)	0 (−0.6, 0.6)	0.691

**Figure 2:**
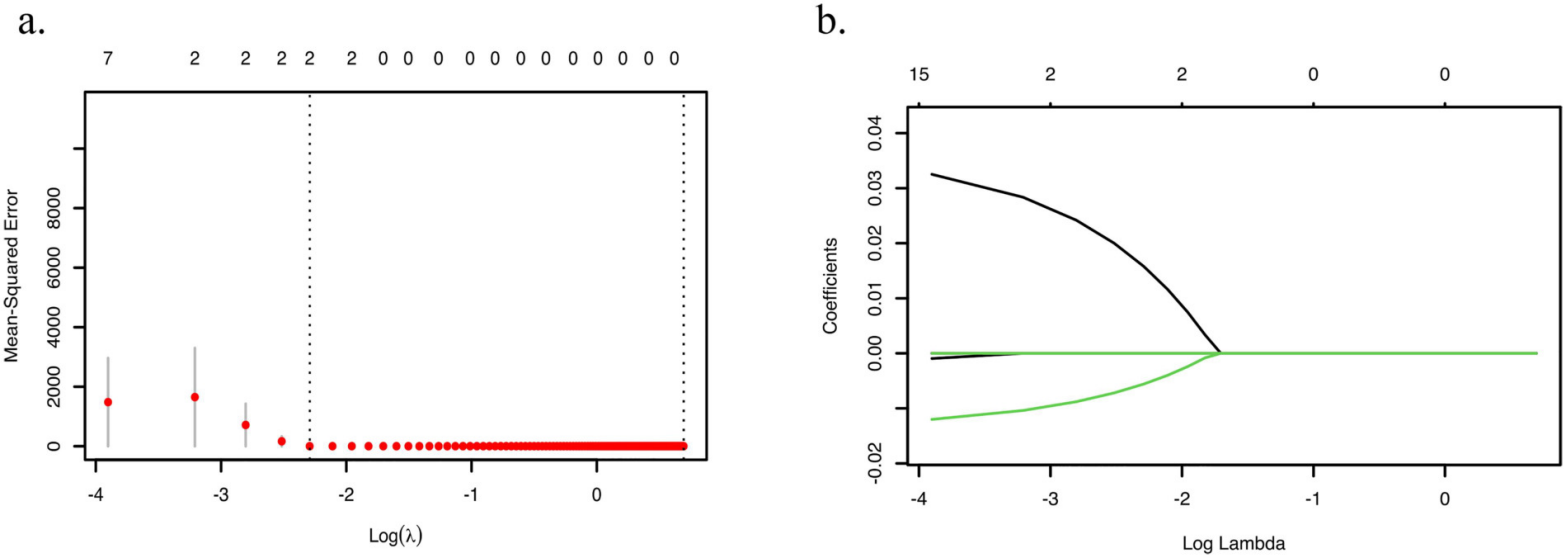
Hyperparameter tuning process of the lasso regression model. **(a)** Lasso penalty strength grid and cross-validation error estimation. **(b)** Optimal λ selection.

Using historical meteorological and hospital admission data, we developed Lasso regression, Random Forest (RF), and Support Vector Regression (SVR) models to predict daily AMI incidence. As illustrated in Figure 2, the Lasso regression implementation followed a rigorous workflow: (1) feature standardization; (2) 5-fold cross-validated grid search over logarithmically spaced *λ* values (*α* = 1); (3) optimal regularization parameter selection (*λ*_min = 0.02) minimizing validation mean squared error (MSE). Regularization paths and error curves were visualized to monitor coefficient shrinkage and performance evolution. The final sparse model achieved an optimal bias-variance tradeoff, retaining clinically relevant predictors while suppressing noise covariates.

**Figure 3:**
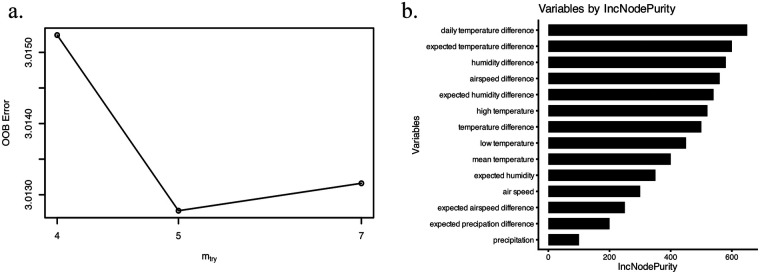
Random forest hyperparameter tuning and feature importance analysis. **(a)** Hyperparameter tuning. **(b)** Feature importance.

As depicted in Figure 3a, automated hyperparameter optimization identified the optimal feature subset size (mtry) for AMI incidence prediction. The tuning protocol systematically evaluated mtry values—initialized at the default setting and explored through 1.5× multiplicative steps—while training 200-tree Random Forest models on predictors (columns 2–16) within the training cohort. This process yielded an optimal mtry value of 5, which subsequently configured the final regression model incorporating all predictors with 200 trees. Predictor importance rankings (Figure 3b) revealed daily temperature difference and expected temperature difference as the two most significant determinants of AMI case number.

**Figure 4:**
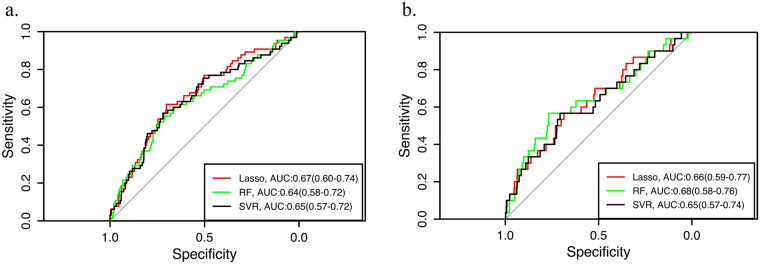
Discriminatory performance of **(a)** 90th and **(b)** 95th percentile thresholds of regression models lasso, random forest, and SVR across decision thresholds.

The Lasso regression model, Random Forest model and SVR model were tested on the test data and the absolute error of them were 1.33,1.36 and 1.31. Initial evaluation at a threshold of 5 demonstrated AUC-ROC values of 0.67 (95% CI 0.60–0.74), 0.64(95% CI 0.58–0.72), and 0.65(95% CI 0.57–0.72), respectively (Figure 4a). Sensitivity analysis at a threshold of 6 yielded AUCs of 0.66 (95% CI 0.59–0.77), 0.68(95% CI 0.58–0.76), and 0.65(95% CI 0.57–0.74), confirming consistent discriminative performance across clinically relevant decision boundaries (Figure 4b).

**Figure 5:**
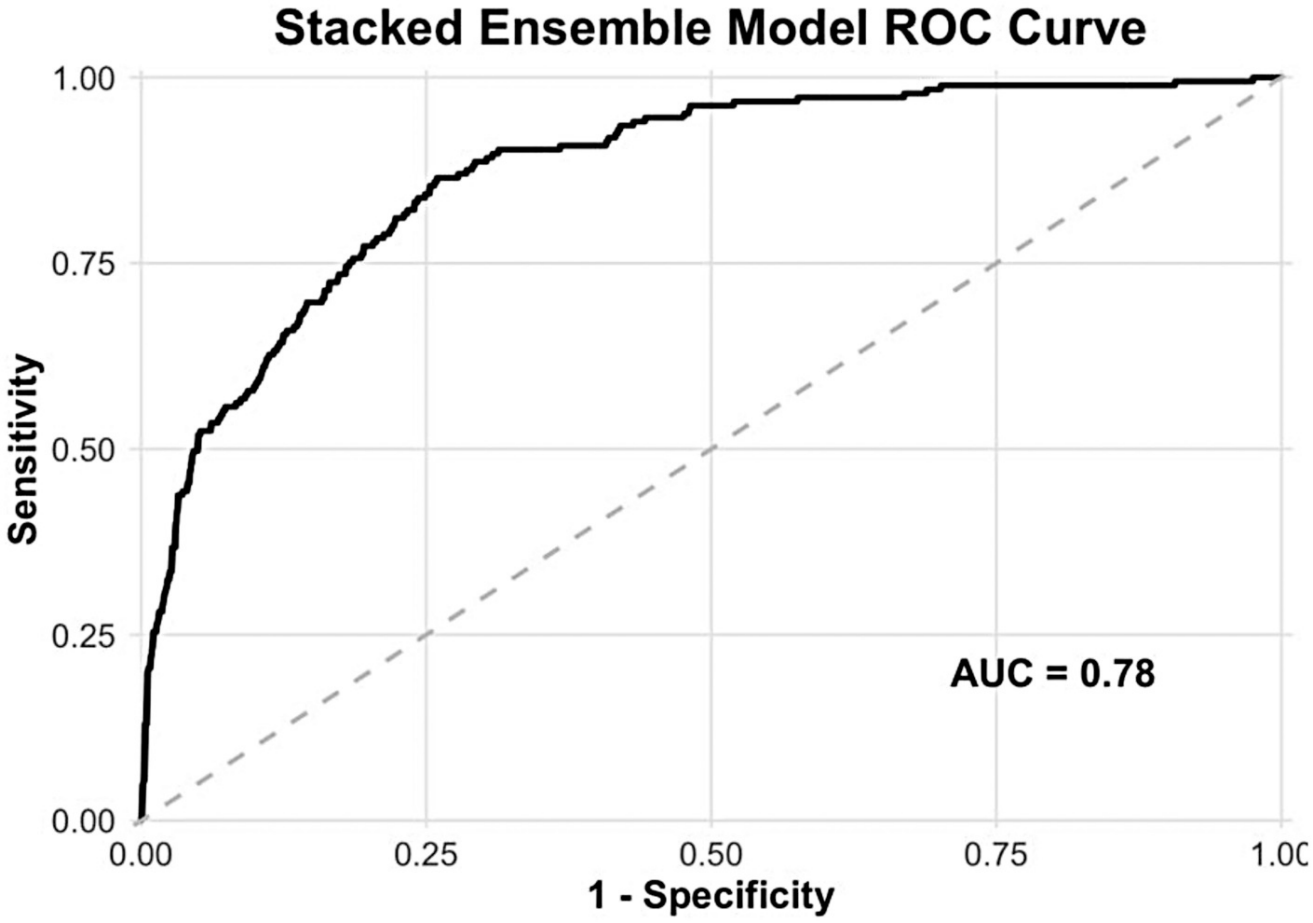
Discriminatory performance of stacked ensemble model.

To enhance predictive performance, we developed a stacked ensemble model integrating the three base learners. This ensemble approach yielded an AUC of 0.78 (Figure 5), representing a meaningful improvement over individual models and demonstrating the added value of combining diverse algorithmic approaches for AMI risk prediction. Based on the Youden's J index maximization principle, the optimal cut-off point of this stacked ensemble model corresponds to a sensitivity of 0.78 and a specificity of 0.80.

## Discussion

This study establishes significant meteorological associations with acute myocardial infarction patterns in the study region, revealing an inverse correlation between daily minimum temperatures and AMI incidence alongside a positive relationship between diurnal temperature variability and high-incidence events. Machine learning algorithms demonstrated effective predictive capacity for identifying high-volume AMI days based on weather parameters, with validation confirming robust discriminative performance across clinically actionable thresholds, supporting the utility of meteorological data in optimizing cardiovascular emergency resource planning.

Substantial evidence confirms meteorological influences on cardiovascular events, with temperature extremes significantly increasing acute myocardial infarction (AMI) hospitalization and mortality (11, 13, 23, 24). Secondary meteorological factors including precipitation may modulate short-term AMI risk (24–27). While prior research indicates linear increases in AMI incidence below 15 °C during cooling periods (28), our analysis specifically demonstrates an inverse correlation between daily minimum temperature and AMI case counts, aligning with established climatic patterns (29). Crucially, multivariable adjustment identified daily temperature difference as an independent predictor of myocardial infarction cluster days (OR 1.38, *p* = 0.002), whereas minimum/maximum temperatures, wind speed, and humidity showed no statistically significant associations after correction for confounding factors.

Meteorological conditions exert profound influences on human physiology, with extreme weather elevating sympathetic tone, blood pressure, heart rate, left ventricular end-diastolic pressure, and myocardial oxygen demand (30). Coronary optical coherence tomography (OCT) analyses demonstrate significantly higher plaque rupture frequency during cold-weather AMI episodes vs. warm-weather events (31), indicating thermally mediated plaque destabilization. Cold exposure induces hemodynamic alterations through pronounced pressor responses (32), exacerbating vascular endothelial injury and potentiating infarction risk. Concomitant heart rate variability (33) generates hemodynamic shear stress fluctuations within coronary vasculature, elevating plaque vulnerability. Furthermore, cold stress disrupts lipid metabolism and cholesterol homeostasis (34), accelerating atherogenic pathways.

While meteorological elements show potential for predicting myocardial infarction incidence in localized populations, extant research predominantly focuses on weather-AMI correlations rather than operational prediction models. This study bridges a critical translational gap by developing machine learning frameworks that convert meteorological data into actionable forecasts for emergency cardiac care allocation. Crucially, our approach introduces a dual predictive paradigm: ① forecasting daily AMI case volumes and ② identifying high-incidence days (exceeding the 90th percentile threshold), enabling proactive resource mobilization at regional chest pain centers.

This study delivers critical operational insights for regional chest pain centers managing AMI patient surges. Effective AMI treatment necessitates coordinated multidisciplinary response systems integrating emergency, interventional cardiology, and critical care teams, alongside dynamic resource allocation. While current protocols prioritize geographic proximity through immediate transfer to nearest facilities, healthcare systems face critical vulnerability during case surges. Crucially, *relative demand spikes*—daily volumes exceeding local capacity thresholds—prolong door-to-balloon times significantly, elevating mortality and complication risks. Our machine learning model enables high-accuracy prediction of high-incidence days (≥90th percentile), empowering proactive resource deployment to maintain therapeutic time targets during demand surges.

While this study successfully identified key meteorological predictors and demonstrated the utility of machine learning in forecasting AMI caseloads, we acknowledge that the employed methodological framework—utilizing established techniques such as multivariable adjustment and Random Forest—is relatively conventional. In this foundational, hypothesis-generating research, we prioritized model interpretability and clinical translatability. Our primary objective was to rigorously establish, within our specific regional context, the existence and predictive value of non-linear relationships between weather patterns and AMI incidence using a robust and widely understood modeling approach. Therefore, the novelty of this work lies in the application of these methods to a unique, long-term regional dataset, yielding the clinically actionable insight that daily temperature variation (*Δ*T) serves as a predominant risk marker. Future research aimed at enhancing predictive performance should indeed explore more complex methodologies, such as deep learning for temporal sequences or ensemble models integrating real-time environmental and clinical data streams.

This study has several methodological constraints: This study has several limitations. This study has several limitations. First, and most importantly, the assessment of meteorological exposure was based on the hospital's location, which may not precisely correspond to the patient's residence or the exact location of AMI onset. It is important to note that, following the clinical principle of prioritizing the nearest facility for acute chest pain, patients are highly likely to be admitted to the closest capable chest pain center. While this practice makes the hospital location a reasonable proxy for the vicinity of the event, it remains an imperfect one. Second, our reliance on hospital admission data fails to capture out-of-hospital AMI deaths, likely leading to an underestimation of true incidence and the resultant burden on medical facilities. Third, the present study adopted same-day meteorological data to predict same-day AMI events, which restricts its practical value for real-time early warning. Although this study focused on exploring the instantaneous association between meteorological factors and AMI, the application of forecasted weather data and the consideration of prediction uncertainty should be addressed in future early-warning systems. Fourth, the predictive models incorporated only meteorological variables; future studies that include other environmental factors, such as air pollution, may improve predictive performance.

## Conclusion

This study demonstrates that specific weather parameters, particularly daily temperature difference and air speed, exhibit statistically significant associations with acute myocardial infarction (AMI) incidence. These findings underscore the potential of integrating meteorologic data—especially temperature variability—into early-warning systems for AMI risk stratification. Future studies should incorporate clinical and demographic covariates to enhance model generalizability.

## Data Availability

The raw data supporting the conclusions of this article will be made available by the authors, without undue reservation.
